# Correlation Between Severity of Obstructive Sleep Apnea and Dental Arch Form in Adults

**DOI:** 10.3390/jcm14207183

**Published:** 2025-10-11

**Authors:** Derek Mahony, Stewart Harding, Chitta Ranjan Chowdhury, Abdolreza Jamilian, Asal Fetrati, Niroj Bhattarai, Peter Borbély, Krisztina Kárpáti

**Affiliations:** 1 Private Practice, Sydney 2045, Australia; derek.mahony@fullfaceorthodontics.com.au (D.M.); bhattarai482@gmail.com (N.B.); 2 Queens Dental Sciences Centre, University of Greater Manchester, Bolton BL3 5AB, UK; s.harding@bolton.ac.uk (S.H.); crcext1@bolton.ac.uk (C.R.C.); 3 The City of London Dental School, University of Greater Manchester, Bolton BL3 5AB, UK; 4 Department of Oral and Maxillofacial Surgery, School of Dentistry, University of Michigan, Ann Arbor, MI, USA; afetrati@umich.edu; 5 Department of Orthodontics and Pediatric Dentistry, Faculty of Dentistry, University of Szeged, 6720 Szeged, Hungary; drborbelypeter@gmail.com (P.B.); drkarpatik@gmail.com (K.K.)

**Keywords:** obstructive sleep apnea, maxillary morphology, intermolar distance, palatal height, craniofacial structure, polysomnography

## Abstract

**Objectives:** This study examines the relationship between maxillary morphology and the severity of obstructive sleep apnea (OSA) in adults, with a focus on intermolar distance (IMD) and palatal height (PH) as predictive factors. **Methods:** A retrospective observational study was conducted at private orthodontic practices in Sydney, Australia. A total of 100 adults (50 OSA patients and 50 controls) were included. OSA diagnosis and severity were confirmed via hospital-based polysomnography. Digital maxillary models were analyzed using the Medit Link software (version 3.2.0; Medit Corp., Seoul, Republic of Korea), and IMD and PH were measured. Statistical analyses included one-way ANOVA and linear regression modeling, with adjustments for age and sex. **Results:** The OSA group exhibited significantly narrower IMD (28.96–35.01 mm) and higher PH (21.68–29.56 mm) compared to the control group (IMD: 36.35–41.50 mm; PH: 18.57–23.51 mm). A negative correlation was observed between IMD and PH across all groups. Linear regression analysis demonstrated a strong association between these craniofacial parameters and OSA severity (R^2^ = 0.76, *p* < 0.001). IMD was negatively correlated with the Apnea-Hypopnea Index (AHI) (*p* = 0.003), while PH was positively correlated (*p* < 0.001). The inclusion of demographic variables did not significantly enhance the predictive model. **Conclusions:** Maxillary morphology associates with OSA severity, with narrower IMD and greater PH linked to higher AHI.

## 1. Introduction

Sleep-related breathing disorders (SRBDs) include a group of conditions characterised by disrupted or abnormal breathing patterns during sleep. These disturbances often lead to poor sleep quality and are associated with multiple systemic health complications [1,2,3]. Among these disorders, OSA is the most prevalent and is characterised by recurrent episodes of partial or complete upper airway obstruction during sleep [4]. The prevalence of OSA in the general adult population has been reported to range from nine to thirty-eight percent, with higher rates observed in older adults. Among men, estimates vary between thirteen and thirty-three percent; while in women, prevalence ranges from six to nineteen percent [5,6,7].

The pathophysiology of OSA involves excessive relaxation of the pharyngeal muscles during sleep, leading to upper airway collapse and intermittent breathing interruptions [8]. These obstructive episodes result in intermittent hypoxemia, hypercapnia, and sleep fragmentation, which reduces the time spent in restorative stages of sleep such as rapid eye movement (REM) sleep. Chronic sleep disruption in OSA has been linked to cardiovascular disease, metabolic dysregulation, and neurocognitive impairments [9,10]. Common symptoms of OSA include loud snoring, choking during sleep, morning headaches, and excessive daytime sleepiness. OSA is influenced by various risk factors, including anatomical features such as a large neck circumference, retrognathia, and enlarged tonsils, as well as obesity, male gender, older age, and sedative use. Additionally, medical conditions like diabetes, hypothyroidism, stroke, and heart failure can contribute to airway instability [1,2,11,12].

Craniofacial morphology has been associated with the risk of OSA by affecting the dimensions and stability of the upper airway. Key anatomical features associated with an increased risk of OSA include mandibular deficiencies, such as retrognathia or micrognathia, which reduce airway space and heighten the likelihood of collapse during sleep. Additionally, a high-arched or narrow hard palate can further restrict airflow. Other contributing factors include an inferiorly positioned hyoid bone, macroglossia, and soft tissue crowding, all of which can compromise airway patency during sleep [1,2,12,13]. 

Most previous investigations into the relationship between dental arch morphology and OSA have concentrated on pediatric populations, leaving relatively limited evidence in adults. Among the adult-focused studies, findings have been inconsistent [14,15,16,17,18,19]. For instance, Irlandese et al. reported that adult OSA patients exhibited narrower and more tapered dental arches compared to control subjects [18]. In contrast, Alqahtani et al. found no significant association between occlusal traits and OSA severity in non-obese adults [14]. Similarly, Ippolito et al. reported no statistically significant relationship between dental arch forms and OSA severity after adjusting for confounding variables such as age, sex, and body mass index (BMI) [15].

These mixed results underscore the ongoing debate regarding the role of dental arch morphology in adult OSA. The present study aims to clarify the relationship between maxillary morphology and OSA severity in an adult cohort. Importantly, OSA diagnosis and severity were confirmed via hospital-based polysomnography. To minimize confounding in assessing the contributions of IMD and PH to OSA, analyses were adjusted for established confounders, including age and BMI. To our knowledge, this is the first study that applies a linear regression model that examines the apnea–hypopnea index (AHI) as a function of both intermolar distance (IMD) and palatal height (PH). An additional model incorporating age and sex as covariates was also evaluated. By jointly assessing the contributions of IMD and PH within a multivariable framework, this study addresses gaps in the current literature and provides novel insights that may help guide personalized diagnostic and therapeutic strategies for OSA management.

## 2. Materials and Methods

### 2.1. Study Design, Sample Size and Study Population

This retrospective observational study was conducted at the authors’ private orthodontic practices in Sydney, Australia. The sample size for this study was determined using G*Power software (version 3.1.9.7; Heinrich-Heine-Universität Düsseldorf, Düsseldorf, Germany) based on the study by Ciavarella et al. [16]. With a 95% confidence level (α = 0.05, two-tailed) and 80% statistical power (β = 0.20), a sample size of 50 participants per group provides the ability to detect a standardized effect size of Cohen’s d ≈ 0.57. A total of 100 adult patients were included in the study, comprising 50 patients diagnosed with OSA (24 females and 26 males) and 50 control subjects without OSA (22 females and 28 males), as presented in Table 1. To minimize selection bias, participants were included chronologically as they presented to the practice from 2020 to 2024, following specific inclusion and exclusion criteria.

Participants were eligible for inclusion if they were 42 ± 5 years old, had a BMI below 30 kg/m^2^, and had fully erupted permanent dentition, excluding third molars. A confirmed diagnosis of OSA using hospital-based polysomnography was required for the OSA group, while the control group required confirmation of the absence of OSA using the same diagnostic method. Exclusion criteria included a history of oral and maxillofacial surgery, prior orthodontic treatment, or dental extractions that could alter arch morphology. Patients with craniofacial syndromes and congenital anomalies were also excluded. Additionally, individuals with cognitive, mental, or physical impairments that could affect participation, as well as those unable to provide written informed consent, were not included in the study.

### 2.2. Diagnostic Procedures

All participants underwent hospital-based polysomnography, which is considered the gold standard for diagnosing OSA. All patients were originally referred to the same respiratory physician, who recorded the presence, absence, and severity of OSA. The severity of OSA was determined based on the Apnea-Hypopnea Index (AHI) score, as outlined by Goyal and Johnson [20]; an AHI score of fewer than five events per hour was considered normal. Mild OSA was defined by an AHI score between 5 and 14 events per hour, moderate OSA by a score between 15 and 29 events per hour, and severe OSA by a score of 30 or more events per hour.

### 2.3. Data Collection and Measurements

Digital maxillary models for each participant were generated using the Medit i700 intraoral scanner (Medit Corp., Seoul, Republic of Korea), which produces high-resolution three-dimensional (3D) models. The 3D models were processed using Medit Link software (Medit Corp., Seoul, Republic of Korea). The following measurements were extracted from the 3D models.

**Figure 1:**
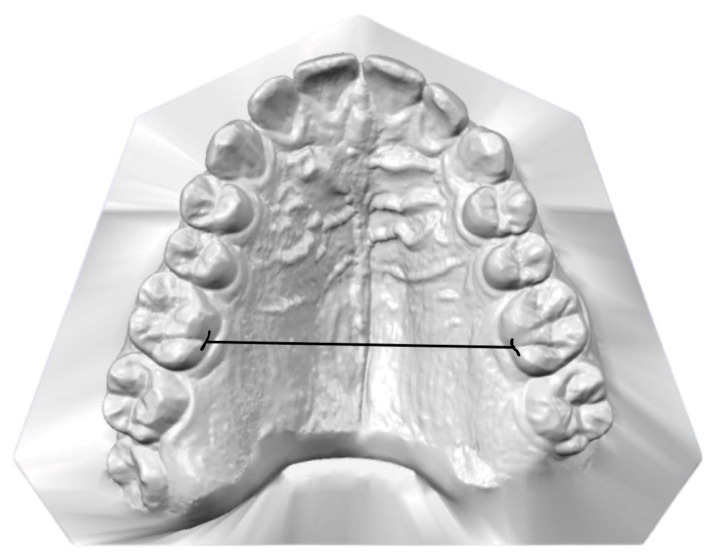
Measurement of IMD: The horizontal line indicates the distance between the palatal grooves of upper first molars.

Intermolar Distance (IMD): This was measured as the linear distance between the palatal grooves of the upper first molars (Figure 1).

**Figure 2:**
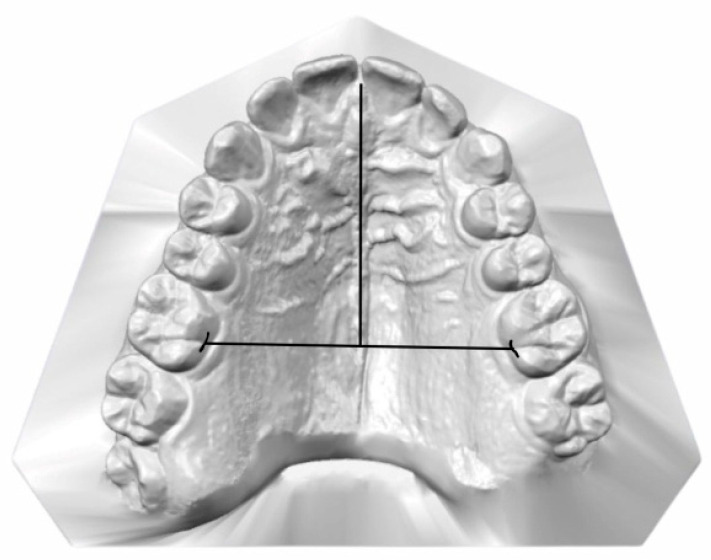
Palatal height measured at the deepest point along the midsagittal plane through the median palatine raphe, perpendicular to the horizontal line defined by the right and left upper first molars.

Palatal Height (PH): Palatal height was determined by establishing a mid-sagittal plane along the median palatine raphe, oriented perpendicular to the horizontal line defined by the right and left upper first molars. Palatal height was measured at the deepest point along this midsagittal plane (Figure 2).

All measurements were performed by a single operator with extensive training in orthodontic assessments. To minimize bias, the operator was blinded to the group allocation of the participants during the measurement process. To validate the accuracy and reproducibility of these measurements, the 3D models were re-evaluated one month after the initial assessment. The intra-class correlation coefficient (ICC) was calculated to assess intra-operator reliability. The average of the two measurements was used in all subsequent analyses.

### 2.4. Statistical Analysis

Statistical analysis was conducted using IBM SPSS Statistics for Windows, Version 29.0 (IBM Corp., Armonk, NY, USA). Descriptive statistics, including means and standard deviations, were calculated for all variables. A one-way analysis of variance (ANOVA) was performed to compare intermolar distance and palatal height between the OSA and control groups. Statistical significance was set at *p* < 0.05.

To further investigate the relationship between maxillary morphology and OSA severity, linear regression analysis was performed with the AHI as the dependent variable and intermolar distance IMD, PH, age, and sex as independent variables.

## 3. Results

The result of ICC analysis for both IMD and PH variables ranged from 0.940 to 0.990, indicating a high level of agreement.

**Figure 3:**
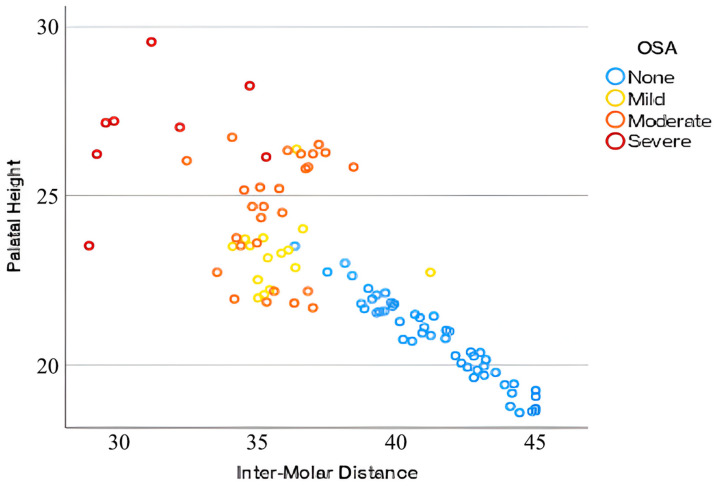
Variation in IMD with PH and distribution of OSA.

In the control group, IMD ranged from 36.35 to 41.50 mm, while the PH ranged from 18.57 to 23.51 mm. As presented in Figure 3, the OSA group exhibited a narrower IMD (28.96 to 35.01 mm) and a greater PH (21.68 to 29.56 mm). These measurements indicate a significant negative correlation between IMD and PH across all groups, with a wider IMD generally associated with a lower PH. On average, the OSA group had a smaller IMD (35.01 mm) compared to the control group (41.50 mm) and a higher PH (24.50 mm vs. 20.72 mm) (Table 1).

**Figure 4:**
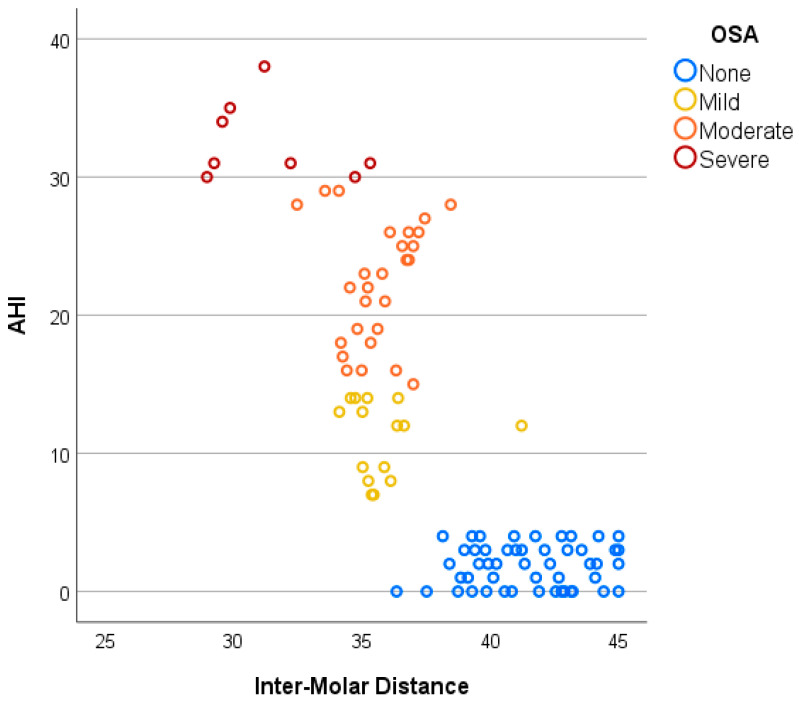
Negative linear relationship between AHI and IMD.

**Figure 5:**
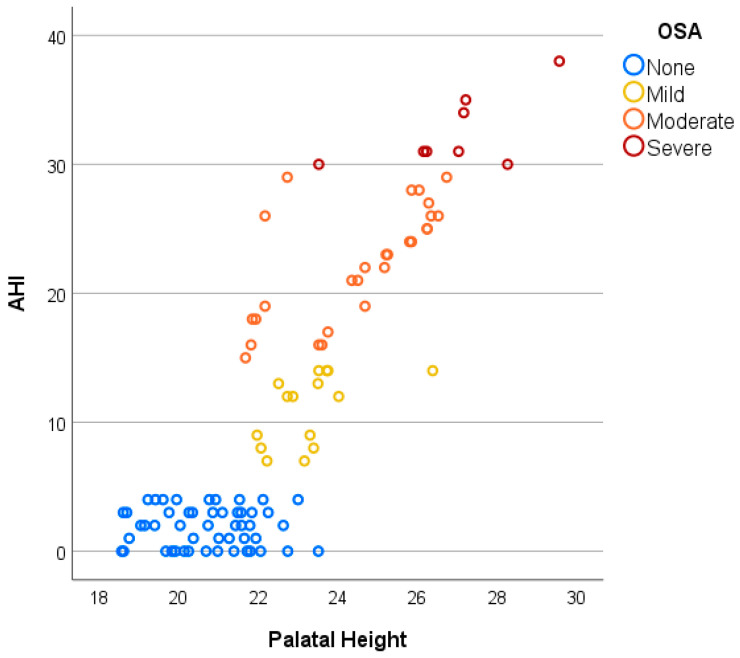
Positive linear relationship between AHI and PH.

**Figure 6:**
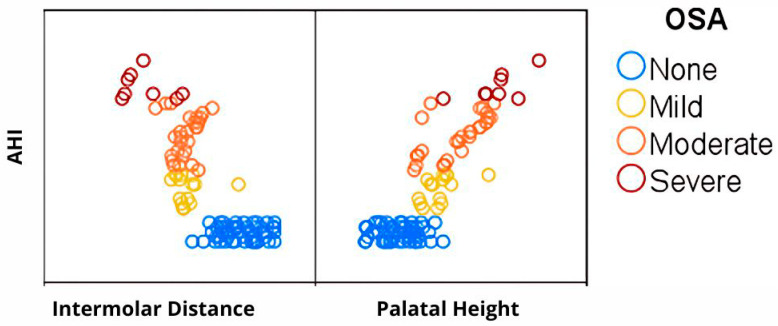
Matrix plot AHI vs. IMD and PH.

Furthermore, with increasing OSA severity, intermolar distance tends to decrease, whereas palatal height increases, as shown in Figure 4 and Figure 5. Patients without OSA exhibit the widest IMD and the lowest PH, followed by those with mild and moderate OSA, while individuals with severe OSA demonstrate the narrowest IMD and the highest PH. Additionally, while non-OSA subjects predominantly present with higher IMD and lower PH values, individuals with OSA demonstrate greater variability in these parameters, particularly in moderate and severe cases, suggesting a more heterogeneous craniofacial profile among those with higher AHI scores (Figure 6).

In a linear regression analysis (Table 2), AHI was modeled as a function of IMD and PH levels. The model revealed a significant association (*p* < 0.001), with the predictors explaining 76% of the variability in AHI (R^2^ = 0.76). Both IMD and PH independently contributed to the model. IMD exhibited a negative association with AHI (*p* = 0.003); a one-unit increase in IMD corresponded to a decrease in AHI by 0.76 units (95% CI: −1.25 to −0.26). PH displayed a positive association with AHI (*p* < 0.001); a one-unit increase in PH resulted in an increase of 2.81 units in AHI (95% CI: 2.03 to 3.59) (Table 2, Table 3 and Table 4).

**Figure 7:**
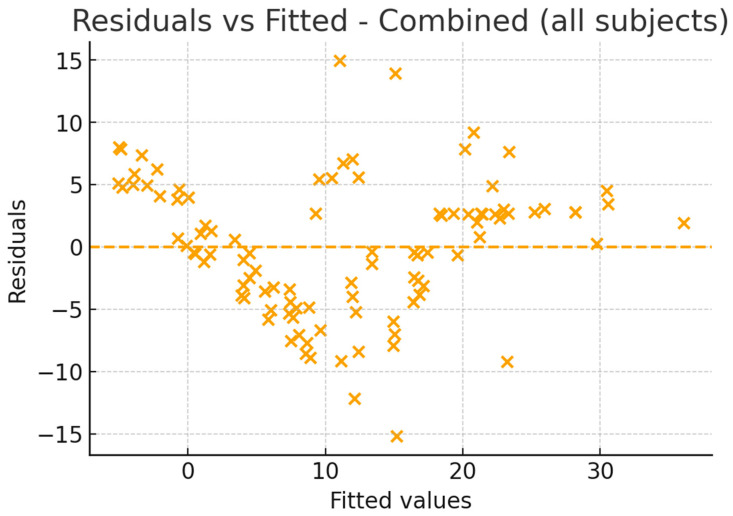
Combined model—Residuals vs. Fitted values.

**Figure 8:**
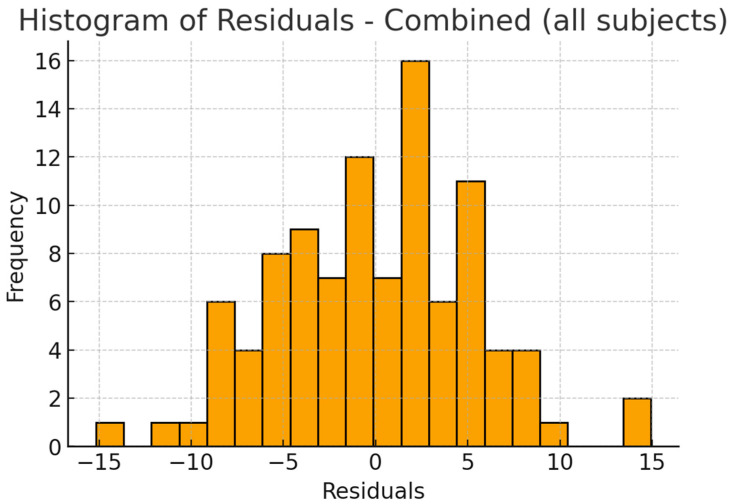
Combined model—Histogram of residuals.

**Figure 9:**
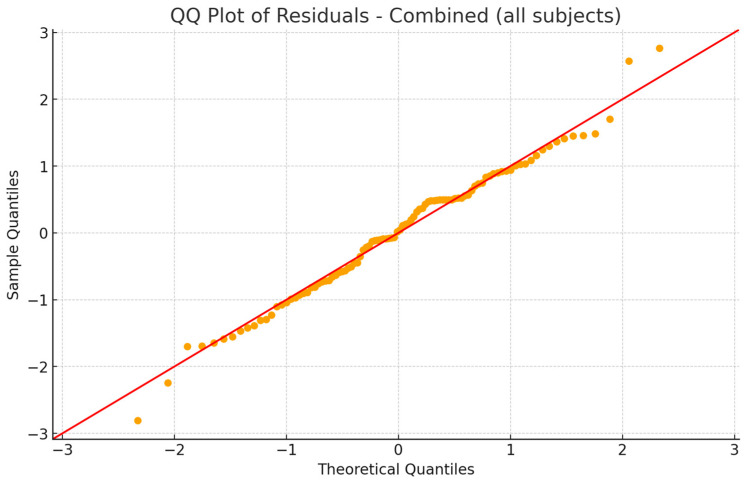
Combined model—QQ plot of residuals.

Residual diagnostics confirmed that the model assumptions were reasonably satisfied. The residuals were symmetrically distributed, with no evidence of heteroscedasticity, and the QQ plot indicated approximate normality. These findings suggest that the regression model is robust and not influenced by outliers (Figure 7, Figure 8 and Figure 9).

Sensitivity analyses further supported the stability of the model. In the full sample (N = 100), IMD and PH together explained 76% of the variance in AHI (R^2^ = 0.76). Restricting the analysis to OSA patients only (N = 50) still yielded a high explained variance (R^2^ = 0.72). Excluding the severe OSA subgroup (N = 92) produced consistent results (R^2^ = 0.74). Across all models, decreased IMD and increased PH remained significantly associated with higher AHI values.

Adding demographic variables (sex and age) to the model slightly improved the explained variance (R^2^ = 0.760), although neither sex nor age achieved statistical significance, indicating their limited impact compared to IMD and PH (Table 5, Table 6 and Table 7).

## 4. Discussion

This study explored the relationship between dental arch dimensions, specifically IMD and PH, and the severity of OSA. The use of a structured methodology, including a well-defined participant age range (42 ± 5 years) and careful control of confounding variables such as BMI and sex, supports the credibility of the findings.

With the sample size of 100 patients (50 OSA and 50 controls), the study had sufficient statistical power to support reliable comparisons. Results revealed a significant negative correlation between IMD and PH, with wider dental arches generally associated with lower OSA severity. Patients without OSA tended to exhibit the highest IMD and lowest PH, whereas those with mild to severe OSA demonstrated greater variability in these measurements. These findings suggest that structural craniofacial differences may be associated with the severity of OSA.

The linear regression model demonstrated that IMD and PH together accounted for over three-quarters of the variability in AHI scores (R^2^ = 0.76, *p* < 0.001). Specifically, each unit increase in IMD corresponded to a 0.76 unit decrease in AHI, suggesting that wider dental arches may promote airway stability and reduce OSA risk. Conversely, each unit increase in PH was associated with a 2.81 unit increase in AHI, indicating that a deeper palate may contribute to airway collapsibility and heightened OSA severity.

Given the relatively high R^2^ value of 0.76, residual and sensitivity analyses were conducted to evaluate model robustness and rule out overfitting. The residuals indicated that the linear regression assumptions were satisfied, while sensitivity models restricted to OSA patients only (N = 50; R^2^ = 0.72) or excluding the severe OSA subgroup (N = 92; R^2^ = 0.74) confirmed the stability of the associations. Although the small number of severe OSA cases (n = 8) limits the strength of subgroup-specific conclusions, the consistent findings across sensitivity models support IMD and PH as reliable craniofacial correlates of AHI in this sample.

Interestingly, the inclusion of demographic variables such as age and sex did not significantly enhance the predictive accuracy of the model. This suggests that craniofacial morphology, particularly arch width and palatal height, may play a more direct role in airway stability than previously assumed. While existing OSA research has predominantly emphasized BMI and age as key risk factors, our findings point to craniofacial features as independent contributors to OSA severity.

Several previous studies support these associations. In a study by Johal and Conaghan [21], a statistically significant difference in palatal height was observed between OSA patients and controls; however, no significant difference was found in IMD. Similarly, Kecik’s study demonstrated that patients with OSA exhibited a reduced palatal volume. Moreover, the IMD in the OSA group was significantly smaller compared to the control group [22]. Kang et al.’s findings in a study on adolescents [23] further support our results, demonstrating that nasomaxillary features, including palatal vault angle, correlate with AHI scores. However, in their study, IMD did not show a significant difference between the OSA and control groups. Similarly, Cistulli et al. and Seto et al. [24,25] did not observe significant differences in PH between OSA patients and controls. Nonetheless, consistent with our study, both reported a reduced IMD in individuals with OSA.

Ciavarella et al. [16] demonstrated a statistically significant inverse correlation between the oxygen desaturation index (ODI) and several craniofacial measurements, including palatal height, palatal area, and maxillary length; again, supporting the idea that narrower structures are linked to reduced airway volume and greater OSA severity.

Our study also indicated a relationship between IMD, PH, and OSA severity, reflecting the structural discrepancies identified by Seto et al. [25]. However, while they reported no significant difference in palatal height between groups, we observed a negative correlation between IMD and PH across varying OSA severities. This discrepancy may be attributed to differences in measurement methodologies or demographic factors, as Seto et al. noted slight variations in age and sex distribution between their groups.

Our results align with those of Nainan et al. [26], showing a similar pattern, in which male participants exhibited greater OSA severity, consistent with existing literature reporting a higher prevalence among men.

While existing literature frequently reports a relationship between age and OSA severity, our study, which standardized age at 42 ± 5 years, did not reveal a significant association. This may be due to the limited age range, which reduced variability and may have obscured broader age-related trends. Furthermore, age likely interacts with other factors such as anatomical features or comorbidities; for example, younger individuals may develop severe OSA due to obesity or craniofacial anatomy, while older adults may experience varying severities based on overall health status. These findings suggest that although age is a recognized factor in OSA, its impact may be less pronounced within a more homogeneous sample. Further research involving a broader age range is needed to fully understand the interplay between age and OSA severity.

By quantifying the impact of IMD and PH on AHI, our study supports the integration of craniofacial assessments into OSA evaluations. Incorporating these parameters into routine dental and orthodontic examinations may facilitate earlier identification of patients at risk, improve risk stratification, and guide more individualized treatment planning. Our results suggest that arch width and palatal height may play a more direct role in airway stability than previously assumed. Given that maxillary constriction may not always present as a crossbite, establishing normative values for the palatal height-to-width ratio based on functional and aesthetic parameters is essential for accurate diagnosis and effective treatment planning. Previous studies have also suggested that rapid maxillary expansion and surgically assisted rapid maxillary expansion may be beneficial in OSA management by increasing nasal cavity volume, reducing airway obstruction, and improving tongue posture [27,28,29,30,31,32,33].

In conclusion, this study highlights the association between dental arch morphology and the severity of OSA. These findings support the potential value of incorporating craniofacial assessments into routine OSA evaluations and considering maxillary expansion techniques in treatment planning.

This study has several limitations. Although the overall sample size was adequate, subgroup distribution was uneven, with only eight patients in the severe OSA group, reducing statistical power and limiting the reliability of subgroup-specific conclusions. The analyses were adjusted only for age and sex, without accounting for other potential confounders such as body mass index, ethnicity, dental arch anomalies, or broader craniofacial characteristics. In addition, the use of a private practice sample may introduce referral bias, which could limit generalizability. Although residual diagnostics and sensitivity analyses confirmed the robustness of the regression models, the relatively high R^2^ values suggest that the findings should be validated in larger, more diverse cohorts to ensure reproducibility. Future studies with larger, more balanced samples and more comprehensive adjustment for covariates are warranted to validate and extend these results.

## 5. Conclusions

This study indicates an association between craniofacial morphology, particularly palatal height and inter-molar distance, and the severity of obstructive sleep apnea. Considering these measures during clinical assessment may enhance risk evaluation for OSA and support the development of more tailored management approaches.

## Figures and Tables

**Table 1:** Demographic data and measurement results.

Groups	Total	OSA Severity	F	M	Age (Years) Mean (SD)	IMD (mm) Mean (SD)	PH (mm) Mean (SD)
***Control***	50		22	28	41.14 (3.12)	41.50 (2.37)	20.72 (1.25)
***OSA***	50		24	26	40.77 (3.23)	35.01 (2.29)	24.50 (1.93)
		*Mild*	8	7		35.83 (1.66)	23.74 (1.07)
		*Moderate*	15	12		35.62 (1.37)	24.48 (1.71)
		*Severe*	1	7		31.39 (2.49)	26.89 (1.75)

**Table 2:** Model Summary: IMD and PH as Predictors of AHI.

Model	R	R^2^	Adjusted R^2^	Std. Error of Estimate
**1**	0.871	0.759	0.754	5.489

**Table 3:** ANOVA: Overall Model Fit.

Source	Sum of Squares	df	Mean Square	F	Sig.
**Regression**	9192.87	2	4596.44	152.57	<0.001
**Residual**	2922.29	97	30.13		
**Total**	12115.16	99			

**Table 4:** Coefficients: Impact of IMD and Palatal Height on AHI.

Predictor	B	Std. Error	β (Beta)	t	*p*-Value	95% CI (Lower–Upper)
**(Constant)**	−23.433	17.564	—	−1.334	0.185	−58.29 to 11.43
**IMD**	−0.755	0.248	−0.271	−3.049	0.003	−1.25 to −0.26
**PH**	2.810	0.393	0.634	7.145	<0.001	2.03 to 3.59

**Table 5:** Model Summary with Demographics (Age and Sex).

Model	R	R^2^	Adjusted R^2^	Std. Error of Estimate
**2**	0.872	0.760	0.750	5.535

**Table 6:** ANOVA: Extended Model Including Age and Sex.

Source	Sum of Squares	df	Mean Square	F	Sig.
**Regression**	9204.21	4	2301.05	75.10	<0.001
**Residual**	2910.95	95	30.64		
**Total**	12,115.16	99			

**Table 7:** Coefficients: Extended Model with Age and Sex.

Predictor	B	Std. Error	β (Beta)	t	*p*-Value	95% CI (Lower–Upper)
**(Constant)**	−20.752	19.177	—	−1.082	0.282	−58.82 to 17.32
**IMD**	−0.745	0.250	−0.267	−2.977	0.004	−1.24 to −0.25
**PH**	2.825	0.398	0.637	7.102	<0.001	2.04 to 3.62
**Age**	−0.077	0.190	−0.020	−0.405	0.687	−0.45 to 0.30
**Sex**	−0.497	1.114	−0.022	−0.446	0.657	−2.71 to 1.72

## Data Availability

The datasets used and/or analyzed during the current study are available from the corresponding author on reasonable request.
